# Effect of online game policy on smartphone game play time, addiction, and emotion in rural adolescents of China

**DOI:** 10.1186/s12888-023-05325-3

**Published:** 2023-11-08

**Authors:** Qiongyao Yang, Han Wang, Hongye Wu, Wei Li, Yueyue Zhang, Yitan Yao, Xiaoping Yuan, Chuanchuan Chen, Yue Wang, Yongjie Zhong, Wenhui Wang, Miaomiao Zhang, Yating Yang, Huanzhong Liu, Kai Zhang

**Affiliations:** 1 Department of Psychiatry, Chaohu Hospital of Anhui Medical University, Hefei, 238000 China; 2 Anhui Psychiatric Center, Anhui Medical University, Hefei, China; 3 School of Mental Health and Psychological Sciences, Anhui Medical University, Hefei, China; 4 Department of Psychiatry, The Fourth People’s Hospital of Maanshan, Maanshan, China

**Keywords:** Adolescent, Emotions, Gaming disorder, Policy, Smartphone addiction

## Abstract

**Background:**

Smartphone game addiction has emerged as a major public health problem in China and worldwide. In November 2019 and August 2021, the National Press and Publication Administration of China implemented two increasingly strict policies, as a means of preventing smartphone game addiction in adolescents aged 18 or below. This study aimed to analyze the effect of the policies on smartphone game play time, addiction, and emotion among rural adolescents in China.

**Methods:**

We sent the questionnaire to rural adolescents through the online survey tool Questionnaire Star, a professional online survey evaluation platform. The questionnaire included demographic data, smartphone use survey, smartphone game addiction and emotion evaluation scales. The Smartphone Addiction Scale-Short Version (SAS-SV) measured adolescents’ smartphone game addiction. The Short Version of UPPS-P Impulsive Behavior Scale (SUPPS-P) and Social Anxiety Scale for Children (SASC) measured emotion. According to SAS-SV score, the enrolled rural adolescents were divided into addiction group and non-addiction group. The *t*-test, Chi-square test, and repeated measure ANOVA assessed the effect of the policies on adolescents’ smartphone game addiction and emotion.

**Results:**

Among enrolled 459 rural adolescents with a mean age of 14.36 ± 1.37years, 151 (32.90%) were in the addiction group and 308 (67.10%) were in the non-addiction group. Adolescents in the addiction group were older, more male, and higher grade. There were time and group effects between the two groups in playtime. After a year of policies implementation, the weekly game time dropped from 3.52 ± 1.89 h to 2.63 ± 1.93 h in the addiction group and from 2.71 ± 1.75 h to 2.36 ± 1.73 h in the non-addiction group. There were also time and group effects in SAS-SV and SASC scores, but not for SUPPS-P score. In the addiction group, the SAS-SV score dropped from 41.44 ± 7.45 to 29.58 ± 12.43, which was below the cut-off value for addiction, and the level of social anxiety was consistently higher than non-addiction group.

**Conclusions:**

The playtime of rural adolescents spent on smartphone games has decreased significantly due to the restriction of the policies rather than the lack of addiction or social anxiety. The policies had practically significant effects in reducing smartphone game play time for rural adolescents in China.

## Background

With the rapid development of technology, the Internet has gradually penetrated all aspects of people’s work and life. According to the China Internet Network Information Center (CINIC) data, as of June 2022, the number of Chinese netizens was 1.051 billion, with 10-19-year-old netizens accounting for 13.5%, and the average weekly Internet time of netizens was 29.5 h [1]. The Internet provides great convenience for learning and leisure, but smartphone overuse or gaming disorder has significant consequences for adolescents. Prolonged use of electronic device was found to be related to physical discomfort, including eye discomfort, musculoskeletal discomfort (wrist, neck, shoulder and back), obesity, sleep deprivation and insufficient physical activity [2–6]. Gaming disorder may lead to several negative mental health problems such as depression, social anxiety, stress, suicide ideation and substance abuse [2, 6]. In addition, studies have reported that students with gaming disorder had poorer academic achievement. This may be explained by poor time management resulting in most of the time being spent on games and poor sleep quality leading to a lack of in-class concentration [7–9].

At present, whether smartphone addiction meets the criteria for addiction is controversial [10], while the International Classification of Diseases (11th Revision) formally defined gaming disorder as addictive behavior in May 2019 [11]. The problem of adolescents addicted to online games has emerged as serious public health problem. However, the etiology of gaming disorder is not fully understood. Some researchers have suggested that the incidence of addictive behaviors in adolescents increases due to the immaturity of cognition and brain development [12]. Multiple protective and risk factors have been considered to be associated with gaming disorder [13]. Self-control, positive parent–adolescent relationship, high levels of school connectedness are protective factors [13, 14]. Impulsivity, maladaptive cognitions and motivations, hostility, deviant peer affiliation, family conflicts, school bullying are positively correlated with gaming disorder [2, 13–15].

The prevalence of gaming disorder varies widely due to the lack of standard definition and heterogeneity in demographics and research methodology. It was reported that the overall prevalence of gaming disorder was 3.3% in general populations [16], while in adolescents it was 4.6% [17], indicating that the prevalence of gaming disorder among adolescents is higher. Gaming disorder is more prevalent in Asian countries, a meta-analysis conducted in 2022 that calculated the prevalence of gaming addiction in East Asia as 12% [18]. A survey of participants from 34 provinces in China showed that the prevalence of gaming disorder among adolescents was 17.0% [2], and the prevalence was higher among adolescent males than females (19.2% versus 7.8%) [19]. It was reported that there was a gap between urban and rural Internet use among Chinese minors [20, 21]. Compared with urban areas, the proportion of mobile phone dependence was higher in rural students, while the proportion of Internet time restricted by parents was lower. The reported prevalence of Internet addiction among left-behind children was higher than that of non-left-behind children due to the lack of parent-child communication and parental supervision [22].

In response, governments around the world have taken regulatory measures to reduce the time of children and adolescents on video games, and those depend on the values and policy goals of various governmental departments [23–25]. In the Western world, video game-related regulations are mainly limited to the rating systems evaluating content and age-appropriateness, such as the Pan European Game Information (PEGI) rating system used in Europe and the Entertainment Software Rating Board (ESRB) used in North America [23]. Compared with the policies implemented by Western countries, Asian countries have clear regulations and mainly aimed at adolescents [23, 24]. The policies limiting the availability of the game include Shutdown system implemented by Thailand, Vietnam, South Korean, China, Selective Shutdown Policy in South Korea, anti-online game addiction system in China, etc. More specifically, for instance, the ‘Juvenile Protection Act’, also known as the Cinderella Law, prohibited individuals under the age of 16 from playing games between 12 midnight and 6 am in South Korean in 2011, which was formally abolished due to it was outdated and basically ineffective in 2021 [25, 26].

In mainland China, governmental regulation of play time is consistent. Drawing on international experience and based on China’s national conditions, the Chinese government has adopted a series of policies to prevent minors from becoming addicted to online games. The National Press and Publication Administration of China (NPPA), one of the agencies directly under The State Council, is in charge of the administration of press and publication and copyright throughout the country. Since 2005, NPPA has organized and formulated the Development Standards and the Real-Name Authentication Scheme of Online Game Anti-Addiction System [27]. In November 2019, NPPA issued a notice to prevent adolescents aged 18 or below from becoming addicted to online games. The policy emphasized the strict implementation of real-name registration and logins, and online game companies could provide no more than one and a half hours of service to minors on ordinary days, with the limit set at no more than three hours on official holidays [28]. The Law of the People’s Republic of China on the Protection of Minors (2020 Revision) put forward protection measures against minors’ Internet addiction, such as online game service providers shall, in accordance with the relevant regulations and standards of the State, classify game products, make age-appropriate warnings, and take technical measures to prevent minors from accessing inappropriate games or game functions (Article 75, paragraph 3) [29]. In December 2020, Online Game Age- Appropriate Tip officially entered the trial stage and provided three different age marks: green 8+, blue 12 + and yellow 16+. For different age levels, the implementation of different game systems, play time, game payment and other operations [30]. In August 2021, NPPA issued stricter regulations to prevent gaming addiction. New regulations required online game companies to allow minors to play only from 8 pm to 9 pm on Fridays, weekends, and official holidays. Press and publication administrations at all levels shall strengthen supervision and deal with companies that fail to put measures in place [31].

There are a large number of left-behind children in rural China, and guardians cannot effectively restrict children’s use of smartphones, which aggravates their addiction to smartphones. At present, smartphone management is a common problem. There are few studies on smartphone game addiction among children in rural areas, and it is imperative to know whether anti-addiction policies reduce the use of smartphones by these children. The main objective of this study was to explore the relationship between anti-addiction policies and smartphone gameplay time, addiction and emotion among rural adolescents in China.

## Methods

### Study participants and data collection

A questionnaire was distributed to rural adolescents through the online survey tool Questionnaire Star starting in September 2021 to collect relevant data, and follow-up visits were conducted in March and September 2022, respectively. The questionnaire includes demographic information, smartphone use characteristics and scale assessments. Informed consent was obtained from the legal guardians of minors involved in the study. Adolescents aged 10–18 who had played smartphone game once or more were included in the study. Exclusion criteria included never having played smartphone game, under 10 years old, over 18 years old, not willing to participate in the study. This study was approved by the ethical review board of Chaohu Hospital of Anhui Medical University, which conformed to the principles embodied in the Declaration of Helsinki.

### Assessment instruments

#### Smartphone addiction scale-short version (SAS-SV)

The SAS-SV is a short version that contains only 10 items with a 6-point Likert scale (1: ‘‘*strongly disagree*’’ and 6: ‘‘*strongly agree*’’) to evaluate smartphone addiction by self-reporting. Compared with SAS, the SAS-SV provides a cut-off value to evaluate the level of addiction and treatment effect, which is better as an appropriate tool for evaluating smartphone addiction in adolescents. The Cronbach’s alpha of the SAS-SV is 0.91 [32].

#### Short version of UPPS-P impulsive behavior scale (SUPPS-P)

The SUPPS-P consists of 20 items assessing five distinct facets of impulsivity, with 4 questions in each dimension, including negative urgency (α = 0.78), lack of premeditation (α = 0.85), lack of perseverance (α = 0.79), sensation seeking (α = 0.74), and positive urgency (α = 0.78). A 4-point Likert scale is used for scoring, and some items are coded in reverse, with higher total scores indicating higher impulsivity. The SUPPS-P is considered a valid and reliable alternative to the original UPPS-P [33].

#### Social anxiety scale for children (SASC)

The SASC consists of 10 items by self-report measure with two factors. Factor 1 is labeled fear of negative evaluation (FNE) with 6 items, and factor 2 is labeled social avoidance and distress (SAD) with 4 items. Anxiety is significantly correlated with FNE and SAD factors. The scores range from *never true* (0) to *sometimes true* (1) to *always true* (2), and several items indicate higher levels of anxiety with lower scores. The standardized alpha reliability coefficient is 0.76 for SASC, and the test-retest reliability is 0.67 [34].

### Statistical analysis

The mean and standard deviation (SD) for quantitative variables and percentage for categorical variables were used to describe the characteristics of participants. The *t*-test, Chi-square test, and repeated measure ANOVA were used to compare the differences between the two groups to assess the effect of the policies on adolescents’ smartphone game addiction and emotions. All statistical analyses were analyzed using SPSS software (version 16.0), and statistical significance was set at a level of two-sided p < 0.05.

## Results

### Demographic characteristics of all enrolled rural adolescents

A total of 459 adolescents completed the study with an average age of 14.36 ± 1.37. Participants were divided into addiction and non-addiction groups based on SAS-SV scores (over 31 points for males, over 33 points for females). There were 151 (32.9%) participants in the addiction group with an average age of 14.58 ± 1.26(minimum,12; maximum,17), and 308 (67.10%) in the non-addiction group with an average age of 14.26 ± 1.40(minimum,11; maximum,18). There were statistical differences in gender and grade distribution between the two groups. Most adolescents came from two-parent families (86.27%), and the proportion of only children was low (20.92%). No significant difference was found in who they lived with or their parents’ educational background between the two groups. The main characteristics of participants are presented on Table 1.

**Table 1:** Demographic data of all enrolled rural adolescents

		Addiction group (n = 151)	Non-addiction group (n = 308)	t/χ^2^	P
Age (years)		14.58 ± 1.26	14.26 ± 1.40	2.34	0.02
Gender					
	Male	93 (61.59%)	148 (48.05%)	7.45	0.01
	Female	58 (38.41%)	160 (51.95%)		
Grade				19.01	0.00
	6	19 (12.58%)	74 (24.03%)		
	7	22 (14.57%)	43 (13.96%)		
	8	43 (28.48%)	111 (36.04%)		
	9	67 (44.37%)	80 (25.97%)		
Only one child				1.17	0.28
	Yes	36 (23.84%)	60 (19.48%)		
	No	115 (76.16%)	248 (80.52%)		
Family model				1.77	0.41
	Two-parent families	127 (84.11%)	269 (87.62%)		
	Single-parent families	14 (9.27%)	26 (8.47%)		
	Redouble parents	10 (6.62%)	12 (3.91%)		
Living with whom				2.22	0.70
	Father	57 (21.92%)	105 (19.81%)		
	Mother	63 (24.23%)	114 (21.51%)		
	Grandparents	84 (32.31%)	197 (37.17%)		
	Brothers/Sisters	42 (16.15%)	88 (16.60%)		
	Others	14 (5.38%)	26 (4.91%)		
Father’s educational background				1.48	0.48
	Primary school	34 (22.52%)	71 (23.20%)		
	Junior high school	105 (69.54%)	200 (65.36%)		
	High school and above	12 (7.94%)	35 (11.44%)		
Mother’s educational background				2.90	0.23
	Primary school	54 (35.76%)	135 (43.83%)		
	Junior high school	81 (53.64%)	141 (45.78%)		
	High school and above	16 (10.60%)	32 (10.39%)		

### Comparison of smartphone use habits between the two groups

The survey found that 54.90% of rural teenagers had their own mobile phones. There were no significant differences in smartphone use habits between the two groups. They often used mobile phones to watch videos, make calls, learning, play games and so on. 87.15% of the adolescents had played mobile games, and more than half of them played mobile games for the first time in grade 3–6 in primary school. Table 2 shows the specific smartphone usage habits among the two groups of participants.

**Table 2:** Comparison of smartphone use habits between the two groups

	Addiction group (n = 151)	Non-addiction group (n = 308)	χ^2^	P
Did you have your own smart phone?			3.93	0.048
Yes	83 (54.97%)	139 (45.13%)		
No	68 (45.03%)	169 (54.87%)		
What activities do you often use your phone for?			8.16	0.09
Call	83 (21.61%)	183 (25.85%)		
Watching video	101 (26.31%)	179 (25.28%)		
Learning	68 (17.71%)	153 (21.61%)		
Play games	77 (20.05%)	109 (15.40%)		
Others	55 (14.32%)	84 (11.86%)		
Have you played mobile games			2.58	0.11
Yes	137 (90.73%)	263 (85.39%)		
No	14 (9.27%)	45 (14.61%)		
When was the first time you played a mobile game			3.71	0.30
Kindergarten	4 (2.68%)	17 (5.94%)		
Grade 1–3	33 (22.15%)	49 (17.14%)		
Grade 3–6	83 (55.71%)	168 (58.74%)		
Junior middle school	29 (19.46%)	52 (18.18%)		

### Effects of the policies on playtime and emotion for the two groups

Table 3 summarizes the changes in smartphone use time and mood of two groups before and after the implementation of the policies. The total number of participants who completed all assessments was 459. There were time and group effects between the two groups in play time. The addiction group spent more time in smartphone game than the non-addiction group at a year ago. With the implementation of the policy, the time spent on smartphone game decreased significantly in both the two groups. Moreover, there is no significantly difference in play time between the two groups now. There were time and group effects between the two groups in the SAS-SV and SASC scores. The SAS-SV scores of the addiction group were significantly higher than those of the non-addiction group, and the scores of the addiction group decreased significantly after the policy was implemented. The SASC score of the addiction group was always higher than that of the non-addiction group, and only the decrease in the non-addiction group was significant. Based on the SUPPS-P, no statistical difference in impulsivity were found between the two groups.

**Table 3:** Play time and emotion evaluation of all participants

		Addiction group (n = 151)	Non-addiction group (n = 308)	F_group×time_	P	η^2^	F_time_	P	η^2^	F_group_	P	η^2^
Play time (h/week)				7.73	0.00	0.081	43.33	0.00	0.337	10.88	0.00	0.110
	A year ago	3.52 ± 1.89	2.71 ± 1.75^**^									
	Half a year ago	3.19 ± 1.86	2.67 ± 1.74^**^									
	Now	2.63 ± 1.93	2.36 ± 1.73									
SAS-SV				116.93	< 0.001	0.204	90.59	< 0.001	0.165	367.63	< 0.001	0.446
	A year ago	41.44 ± 7.45	19.95 ± 7.34^***^									
	Half a year ago	35.32 ± 8.22	20.64 ± 8.78^***^									
	Now	29.58 ± 12.43	20.70 ± 10.67^***^									
SUPPS-P				3.05	0.06	0.007	1.81	0.17	0.004	0.22	0.64	0.000
	A year ago	50.74 ± 9.11	50.36 ± 10.82									
	Half a year ago	50.48 ± 9.17	51.05 ± 10.27									
	Now	49.62 ± 10.83	50.75 ± 10.52									
SASC				0.40	0.62	0.001	6.05	0.01	0.013	9.10	< 0.001	0.020
	A year ago	20.36 ± 4.77	18.98 ± 5.52^**^									
	Half a year ago	20.19 ± 4.67	18.63 ± 5.46^**^									
	Now	19.97 ± 5.36	18.34 ± 5.74^**^									

Abbreviations: SAS-SV, Smartphone addiction scale-short version; SUPPS-P, Short version of UPPS-P impulsive behavior scale; SASC, Social anxiety scale for children. Compare with addiction group, ^**^P < 0.01; ^***^ P < 0.001

## Discussion

The play time of rural adolescents spent on smartphone game has decreased significantly after the implementation of the policies. It was reported that the prevalence of gaming disorder increased for adolescents during the COVID-19 pandemic [35, 36]. The data showed the prevalence of minors playing mobile games was 53.2% in China in 2021, down 3.2% points from 2020 [21]. In 2022, the weekly gaming time of minors was further reduced after the policies were implemented in China, such as 75.49% of minors played less than 3 h per week in 2022 and 67.76% in 2021 [37]. The participants’ mean SAS-SV score was below the cut-off value for addiction after the new regulations had been implemented for one year. It is reasonable for this study to conclude that the policies have practically significant effects in reducing smartphone gameplay time for rural adolescents in China.

The results found that adolescents in the addiction group were older, more male, and higher grades than those in the non-addiction group, which is consistent with previous researches [6, 38]. Boys spend the most time on gaming, but girls were more likely to engage in social media [39]. Study found excessive smart device use for leisure more prevalent than learning among adolescents [3], and minors tend to devote their time to short videos after gaming is restricted [37]. Maladaptive cognitions, psychological features and relevant brain areas were found to be associated with sexual dimorphism and gaming disorder [19, 40]. Such sex differences should be noted in future studies. Some external factors were associated with a higher risk of gaming disorder, including single-parent families, low socio-economic status, low mother’s education level, poor family relationships, excessive use of video games by parents, and physical or verbal abuse exerted by parents [41, 42]. Our study did not find any correlation between family background and gaming disorder, which may be related to the fact that the sample came from rural children in the same area.

However, the result showed that 45.03% of the adolescents in the addiction group did not have their own smartphone. We know that a proportion of adolescents are using family members’ mobile phones or borrowing others’ mobile phones to play games. Grandparents, out of coddling their grandchildren, may give mobile phones to children without restraint. In addition, they may increase the time of smartphone games through some circumvention methods, including registering with the real name of the parents’ ID card, fraudulently obtaining facial recognition from family members, using other relatives, friends and other adults’ identity information, borrowing or buying game accounts to bypass the supervision of the anti-addiction system. At present, accurate identification of minors online is a challenging task.

High impulsivity and low self-control are key risk factors of gaming disorder, and adolescents with high impulsivity may exhibit heightened spontaneous responses to behavioral cues to games, especially among male adolescents [15, 38]. The current study did not find a statistical difference in impulsivity between the two groups, and one reason for this may be that the sample being from a non-clinical background. Social anxiety is reportedly associated with behavioral addictions [43, 44]. Individuals with gaming disorder tend to have less face-to-face interaction because they spend most of their time playing games. Studies showed that social anxiety was lower when interacting online than offline, socially anxious gamers believe that online communication can avoid the distress of face-to-face social interactions, and the negative metacognitions about online gaming played a mediating role in the relationship between social anxiety and gaming disorder [44, 45]. Social anxiety would elevate gratification of Internet gaming, which has also been suggested to result in gaming disorder [46].

The prevention of adolescents’ addiction to online games requires the cooperation of the government, schools, families and enterprises. China is one of the few countries in the world that have national measures to prevent public health hazards of online games. In practice, the truly implementation is conducive to the prevention and treatment of gaming disorder. Internet usage reportedly declined only in the first two years after the Cinderella Law was implemented in South Korea [47], suggesting the importance of translating policies into action and keeping up with the times. In the future, research and development protection procedures should be strengthened, and anti-addiction systems should be continuously upgraded to prevent bypassing supervision. Game companies and platforms should continue to improve social responsibility, strictly implement anti -addiction policies, and provide high -quality and healthy game products. Schools should strengthen publicity and education, carry out various activities, strictly standardize terminal equipment management, promote parents’ performance of monitoring responsibilities, and ensure that primary and secondary school students grow up healthily under a good network environment. Parents should establish a harmonious parent -child relationship with their children and increase their companionship. Additionally, parents should play an exemplary role, guide their children to use the network correctly and limit play time reasonably because study showed restrictive parental mediation produced a boomerang effect, which increasing child-parent conflict and possibly exacerbating addictive use [48].

The study has several limitations. First, the generalization of the results to adolescents may be limited because our study was conducted among rural adolescents, more research based on large samples, urban and rural integration should be conducted to verify those figures. Second, participants smartphone use time was based on self-reporting, without standard records, and time management software is conducive to truly understanding of smartphone usage. Third, the effect of anti-addiction policies is presented indirectly because it is affected by multiple potentially confounding factors. However, it is reasonable to interpret the effectiveness of policies in the context of domestic longitudinal survey reports on Internet use among minors. Taking the Cinderella Law in South Korea as an example, further research should be conducted on whether policies are directly effective and long-term effective. Additionally, this study only explored the effectiveness of the policies and did not further explore the reasons for the high prevalence of gaming disorder among rural adolescents, which is very important for preventing and reducing excessive gaming. Last, common Internet-based addictive behaviors, including Internet addiction, online gaming disorder, online gambling disorder, pornography use, and smartphone use disorder. Research targeting a specific purpose may be conducive to understanding behavioral addiction.

## Conclusions

After the policies of firmly preventing minors from being addicted to online games was implemented, smartphone gameplay time decreased significantly among rural adolescents in China. Due to the high prevalence and adverse effects of gaming disorder, for the healthy growth of adolescents, the anti-addiction system still needs to be upgraded, and parents should strengthen the supervision of adolescents using mobile phones. Adolescents with gaming disorder commonly have emotional problems, healthcare providers should provide psychological intervention. Due to lack of evidence-based clinical interventions, prevention-oriented and comprehensive intervention are important principles in dealing with game disorder.

## Data Availability

The datasets used and/or analyzed during the current study are available from the corresponding author on reasonable request.

## Abbreviations

CINIC: China Internet Network Information Center
NPPA: National Press and Publication Administration of China
SAS-SV: The Smartphone Addiction Scale-Short Version
SUPPS-P: The Short Version of UPPS-P Impulsive Behavior Scale
SASC: The Social Anxiety Scale for Children
